# Berry syndrome: a case report and literature review

**DOI:** 10.1186/s12872-020-01837-y

**Published:** 2021-01-06

**Authors:** Wen-jing Bi, Yang-jie Xiao, Yue-jia Liu, Yang Hou, Wei-dong Ren

**Affiliations:** 1 Department of Ultrasound, Shengjing Hospital of China Medical University, 36# of Sanhao St. Heping District, Shenyang, 110004 China; 2 Department of Radiology, Shengjing Hospital of China Medical University, 36# of Sanhao St. Heping District, Shenyang, 110004 China

**Keywords:** Berry syndrome, Aortopulmonary window, Aortic origin of the right pulmonary artery, Interrupted aortic arch, Case report

## Abstract

**Background:**

Berry syndrome, a rare combination of cardiac anomalies, consists of aortopulmonary window (APW); aortic origin of the right pulmonary artery; interrupted aortic arch (IAA) or hypoplastic aortic arch or coarctation of the aorta; and an intact ventricular septum. There is lack of review articles that elucidate the clinical features, diagnosis, treatment, and outcomes of Berry syndrome. This publication systematically reviews the 89 cases published since 1982 on Berry syndrome.

**Case presentation:**

A 38-year-old woman presented with a loud murmur and cyanosis. Transthoracic echocardiography demonstrated a severely dilated aorta and main pulmonary artery with a large intervening defect. Distal to the APW, the ascending aorta gave rise to the right pulmonary artery. Additionally, a type A IAA, an intact ventricular septum, and a large patent ductus arteriosus were revealed. Computed tomography angiography with 3-dimensional reconstruction confirmed above findings. This is the first report of a patient of this age with Berry syndrome who did not undergo surgery.

**Conclusions:**

Berry syndrome is a rare but well-identified and surgically correctable anomaly. Patients with Berry syndrome should be followed up for longer periods to better characterize long-term outcomes.

## Background

Berry syndrome is characterized by a constellation of abnormalities: aortopulmonary window (APW); aortic origin of the right pulmonary artery (AORPA); interrupted aortic arch (IAA) or hypoplastic aortic arch (HAA) or coarctation of the aorta (CoA); and intact ventricular septum. It is extremely rare, with most cases published as individual reports [1, 2] or in articles describing patients with these abnormalities [3, 4]. Since first described by Berry et al. in 1982 [1], the anomaly has been reported in the English literature in nearly 100 patients. Most were diagnosed in infancy [5, 6], and the surgical procedure was performed immediately. In contrast, our patient was not diagnosed until she was 38 years old. To the best of our knowledge, this is the first report of a patient of this age with Berry syndrome who did not undergo surgery.

**Fig. 1:**
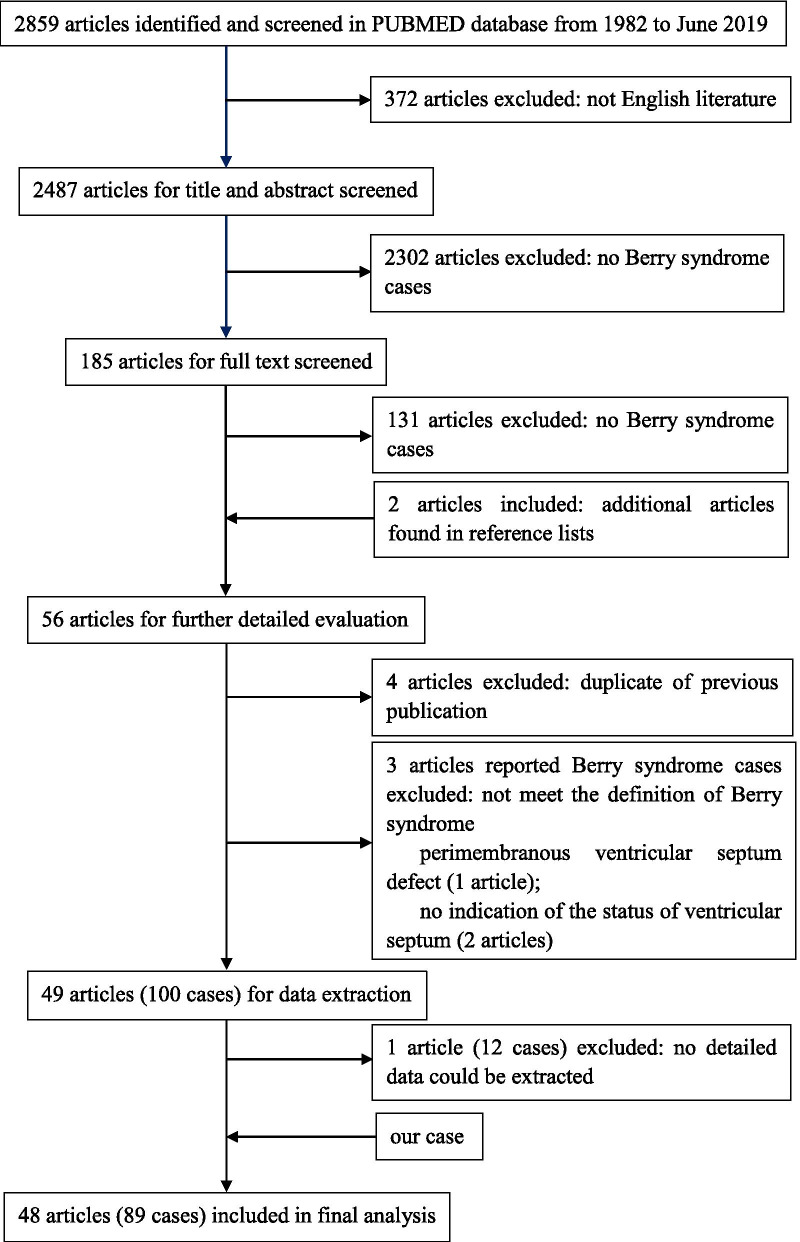
Flowchart of the literature selection process

We present a review and summary of the English literature available regarding this rare anomaly. A PUBMED-based search was conducted using the primary search terms “Berry syndrome”, “aortopulmonary window”, “aortopulmonary septal defect”, “interrupted aortic arch”, “hypoplastic aortic arch”, “coarctation of aorta”, “aortic origin of right pulmonary artery”, and “hemitruncus”. The flow diagram of the study selection process is shown in Fig. 1. Two additional articles were found by checking reference lists [7, 8]. Four duplicate studies were excluded [9–12]. Three articles were excluded because the patients did not meet our definition of Berry syndrome [13–15]. One article was excluded because the data could not be extracted [16]. Our case, combined with those described in the 48 selected articles, comprised a total of 89 evaluable cases [1–8, 17–56].

## Case presentation

**Fig. 2:**
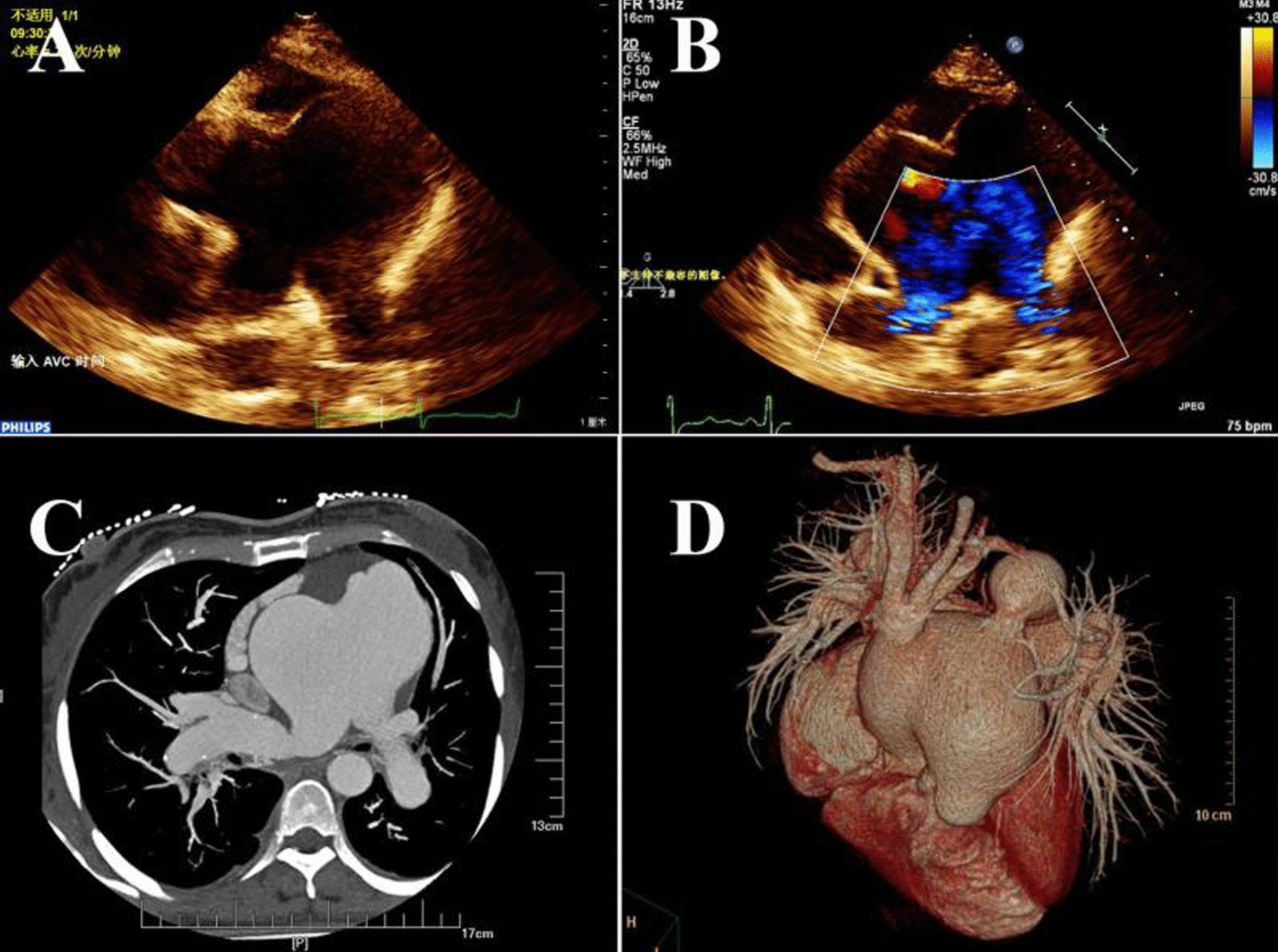
Transthoracic echocardiography demonstrated a severely dilated aorta and main pulmonary artery with a large intervening defect (**a**, **b**). Distal to the APW, the ascending aorta gave rise to the right pulmonary artery (**a**, **b**). Computed tomography showed a type III APW and an AORPA (**c**). Three-dimensional computed tomography revealed a dilated aortic sac, IAA, and the descending aorta formed via a PDA (**d**). AORPA, aortic origin of the right pulmonary artery; APW, aortopulmonary window; PDA, patent ductus arteriosus; IAA, interruption of the aortic arch

A 38-year-old woman received a tentative diagnosis of congenital cardiac disease in childhood. She is a farmer in Liaoning province (not high altitude) with no special lifestyles. She presented in our outpatient for further diagnosis. Physical examination revealed a loud murmur and cyanosis. Electrocardiogram demonstrated normal rhythm and right ventricular hypertrophy with right axis deviation. Echocardiographic study showed a severely dilated right ventricle, with a diameter of 39 mm; the left ventricle was relatively small, with a diameter of 33 mm. The arterial trunk was markedly enlarged (71–105 mm), with a type III APW (Fig. 2a, b) measuring approximately 65 mm. A right pulmonary artery (RPA) originating from the posterior wall of the ascending aorta was also found, while the origin of the left pulmonary artery (LPA) was normal (Fig. 2a, b). The coronary arteries were normally positioned. Additionally, the patient had a type A IAA (distal to the left subclavian artery), an intact ventricular septum, and a large patent ductus arteriosus (PDA) with bidirectional shunts. The patient had severe pulmonary artery hypertension, approximately equal to the systemic blood pressure, and the left ventricular ejection fraction (LVEF) was nearly 53%. Computed tomography angiography with 3-dimensional reconstruction confirmed the large APW opening into the main and right pulmonary arteries, as well as type A IAA supplied by a large ductal artery (Fig. 2c, d). The patient refused to be hospitalized to further evaluate the possibility of surgery, and she was lost to follow-up. Although missing the opportunity to confirm above findings by surgery, a diagnose of Berry syndrome was made.

## Discussion and conclusions

Berry syndrome is an extremely rare disease, accounting for only 0.046% of all reported cases of congenital heart anomalies [52]. It is more common in males (67.1%). Patients commonly presented with one or more clinical symptoms, including cyanosis, respiratory distress symptoms, cardiac murmur, symptoms of congestive cardiac failure, and other systemic symptoms (Table 1). The distribution of age at diagnosis in 81 patients was: 13 (16.0%) diagnosed prenatally, 43 (53.1%) diagnosed before 3 months, 13 (16.0%) diagnosed between 3 months and 1 year; 5 (6.2%) diagnosed between 1 and 12 years, and 2 (2.5%) diagnosed after 12 years; 5 of the patients (6.2%) were diagnosed at autopsy.

**Table 1:** Characteristics of 89 reviewed cases of Berry syndrome

Variable (Number*)	n (%)
Sex (73)	
Male	49 (67.1%)
Premature birth (28)	
Yes	4 (14.3%)
Presence of clinical symptoms	
Cyanosis (52)	21 (40.4%)
Tachypnea/dyspnea/respiratory infection (52)	31 (59.6%)
Cardiac murmur (52)	23 (44.2%)
CHF/poor peripheral perfusion/tachycardia (52)	15 (28.8%)
Poor feeding/lethargy/poor phonation/anuria (52)	10 (19.2%)
Age at diagnosis (81)	
Prenatal	13 (16.0%)
≤ 3 months	43 (53.1%)
3 months-1 year	13 (16.0%)
1–12 years	5 (6.2%)
> 12 years	2 (2.5%)
Autopsy	5 (6.2%)
Diagnostic methods (81)	
Cases used echocardiography	67 (82.7%)
Anomalies recognized by echocardiography (46)	
APW + AORPA + IAA/HAA/CoA	28 (60.9%)
APW + IAA/HAA/CoA	11 (23.9%)
Others	7 (15.2%)
AA morphology (89)	
IAA	83 (93.3%)
CoA	4 (4.5%)
HAA	2 (2.2%)
IAA types (76)	
A	67 (88.2%)
B	9 (11.8%)
C	0 (0)
APW types (71)	
I	2 (2.8%)
II	56 (78.9%)
III	13 (18.3%)
Types of ductus artery (77)	
Patent	76 (98.7%)
Fibrous cord	1 (1.3%)
Types of PDA (31)	
Large	20 (64.5%)
Constricting	11 (35.5%)
Original site of RPA (86)	
Type A (a straddling RPA)	13 (15.1%)
Type B (a completely separated RPA)	73 (84.9%)
Associated cardiac and extracardiac anomalies (68)	
Yes	11 (16.2%)
Chromosomal abnormalities (14)	
Yes	4 (28.6%)
Age at surgery (62)	
≤ 1 month	31 (50.0%)
1–3 months	12 (19.4%)
3 months-1 year	14 (22.6%)
1–12 years	4 (6.5%)
> 12 years	1 (1.6%)
Types of surgery (63)	
One-stage	53 (84.1%)
Two-stage	10 (15.9%)
Postoperative complications	
Aortic arch stenosis (29)	13 (44.8%)
RPA stenosis (29)	17 (58.6%)
Residual APW (29)	1 (3.4%)
Reintervention after surgery (27)	
Yes	13 (48.1%)
Presence of massively dilated vascular sac (64)	
Yes	5 (7.8%)

AORPA, aortic origin of the right pulmonary artery; APW, aortopulmonary window; CHF, congestive heart failure; CoA, coarctation of the aorta; HAA, hypoplastic aortic arch; IAA, interrupted aortic arch; PDA, patent ductus arteriosus; RPA, right pulmonary artery

^*^The total number of cases with available data for analysis

According to the possible embryogenesis described by Berry et al. [1], when the aortopulmonary septum fails to form, a large APW may represent a partial persistence of the common arterial trunk. Affected by this, the sixth pharyngeal arches may fail to form a bifurcation and may attach improperly to the undivided truncal segment. This results in a wide separation between the right and left pulmonary arteries, and in turn, an AORPA is formed. During fetal development, the APW and AORPA could reduce blood flow in the aortic arch, leading to HAA/CoA/IAA. According to our review, most patients with Berry syndrome have type II APW (78.9%). Consistent with previously reported findings [6], the RPA usually arose from the aorta (84.9%), and most cases presented with type A IAA (88.2%).

Associated cardiac and extracardiac anomalies were common. Among 68 patients for whom such detailed information was available, ﻿concomitant cardiac and extracardiac anomalies were diagnosed in 11 (16.2%), including two with an atrial septal defect (one with a small muscular ventricular septal defect and trisomy 13), four with patent foramen ovale, one with abnormalities associated with trisomy 13, one with anomalous origin of the left coronary artery from the main pulmonary artery, one with situs inversus, one with an aberrant right subclavian artery arising from the descending aorta, and one with persistent left superior vena cava. Some authors have reported the important role of neural crest cells in the development of the aortic and pulmonary trunks of the heart [21, 57]. However, the chromosomal abnormalities found in the Berry syndrome cases we reviewed (see Additional file 1) did not seem to be pathogenetically related to a neural crest deformity, which is more likely to be related to a 22q11 gene deletion [57]. To better elucidate the genetic mechanisms underlying the unusual constellation of defects in Berry syndrome, more genetic studies are needed.

Echocardiography is an essential methodology for the diagnosis of complex congenital heart disease. Echocardiography was the initial diagnostic method used in 82.7% of the cases we included. The accuracy rate of echocardiographic diagnosis was 60.9%. In 23.9% of cases, missing recognition of an AORPA resulted in a partially accurate diagnosis. Since AORPA can be readily diagnosed at the time of surgery, it does not increase the difficulty of the surgical procedure or necessitate modification of the therapeutic approach [33]. Thus, echocardiography can provide adequate diagnostic information in most cases, as reported previously [16].

Excluding one patient who was lost to follow-up, the mortality rate for unoperated patients was 100%. The age at death ranged from 1 day to 18 years (median, 1 month). Most unoperated patients died within the first month of life (55.6%). Some of them rapidly developed pulmonary vascular obstructive disease and congestive heart failure [55]. Few unoperated patients may achieve long-term survival, such as the 38-year-old woman we describe in this report. In this case, the arterial trunk was extraordinarily enlarged. On review, we found this distinctive feature in 4 other patients [1, 27, 38, 56], one of whom was not diagnosed until he was 15 years old [1] while other 3 patients underwent surgery before two years old. In Berry syndrome, the arterial trunk has 4 exits (LPA, RPA, PDA, and cranial branches). The LPA and RPA are connected to the lungs. The PDA and cranial branches are connected to the lower and upper body, respectively. Since the PDA was large enough to maintain adequate systemic perfusion to the lower half of the body, a new balance may have been established among the large APW, PDA, and the adaptable ventricles in our case. We speculate that the large arterial trunk may represent a partial persistence of the common arterial trunk and provide a place not only for the full mixing of systemic and pulmonary blood, but also to share volume and pressure overload. This could protect the pulmonary vascular bed and prevent early pulmonary vascular obstructive disease delicately. Thus, these abnormalities (large APW, AORPA, IAA, and large PDA) may have a buffering effect on each other when coexisting with a large arterial trunk, and the affected patients have improved long-term survival. However, our speculation should be confirmed in future studies.

Among all patients with congenital heart disease, 5% to 10% have PDA [58], while almost all of the Berry syndrome patients we included (98.7%) had PDA. The type of PDA was available for 31 cases: 20 patients (64.5%) had a large PDA while 11 patients (35.5%) had constricting PDA. A closed ductus arteriosus or a constricting PDA in a patient with Berry syndrome may predict a poor prognosis. Thus, many patients receive prostaglandin E2 to maintain an open ductus arteriosus until further treatment [8, 47, 51, 53]. On review, more than half of patients diagnosed within 3 months present with a large PDA, while patients diagnosed after 3 months all present with a large PDA. We speculate that the PDA will remain open for a long time in some Berry syndrome patients. In these patients, prophylactic use of prostaglandin may not be necessary before surgery.

The choice of surgical procedure for Berry syndrome remains controversial. ﻿One-stage repair is achieved by closing the APW, connecting the RPA to the main pulmonary artery, and establishing aortic arch continuity. Two-stage repair involves placing surgical pulmonary bands to control pulmonary blood flow at the first stage. After the ventilation status improves, the patient undergoes definitive surgical repair [2]. Recently, one-stage surgical repair has been considered to yield more acceptable outcomes [6]. Among the patients we included, 84.1% underwent one-stage repair while others underwent two-stage repair. The age distribution at surgery was: 31 (50.0%) before 1 month, 12 (19.4%) between 1 and 3 months, 14 (22.6%) between 3 months and 1 year, and 4 (6.5%) between 1 and 12 years; only one patient (1.6%) underwent surgery after 12 years. Surgery age ranged from 0.1 to 204.0 months (median, 1.0 months; interquartile range, 0.5–4.0 months). Regardless, there were no differences in mortality within a month postoperatively (x^2^ = 0, *p* = 1.0) or survival time within the one-year follow-up period (x^2^ = 0.43, *p* = 0.51) between these two groups. For premature and small-for-gestational-age infants, Ghelani et al. [2] advocated two-stage surgery. Premature birth was only reported in four of the Berry syndrome patients we included; of these, 3 underwent two-stage surgery [2, 18, 24], and 1 underwent one-stage surgery [46]. All of these four patients were alive at the times of final follow-up. Given the small sample size and short follow-up period, it is difficult to determine which surgical approach is better.

In pediatric patients younger than three months, the increased pulmonary artery pressure is assumed as mean pulmonary artery pressure exceeded 25 mm Hg [59]. In those older than three months, pulmonary hypertension is defined as mean pulmonary artery pressure exceeded 25 mm Hg [59]. All 19 patients younger than three months, for whom preoperative pulmonary artery pressure was reported, showed increased pulmonary artery pressure. Among 7 patients younger than three months for whom postoperative pulmonary artery pressure was reported, 2 patients (28.6%) showed normal pulmonary artery pressure; 5 patients (71.4%) showed tricuspid regurgitation gradient decreased below 40 mm Hg. All 7 patients older than three months, for whom preoperative pulmonary artery pressure was reported, showed pulmonary hypertension. Among 10 patients older than three months for whom postoperative pulmonary artery pressure was reported, one patient (10%) showed normal pulmonary artery pressure; 9 patients (90%) showed tricuspid regurgitation gradient decreased below 40 mm Hg.

Berry syndrome patients are usually critically ill and need urgent surgery [6]. Because of the large APW and aortic origin of the RPA, most specialists believe that critical pulmonary arterial hypertension appears early after birth [1, 5, 55]. According to some authors, severe pulmonary vascular disease might develop as early as the first 3 months of life due to massive pulmonary blood flow and circulating vasoconstrictors [60]. In our review, almost one-third of the patients were older than 3 months at surgery. With such a delayed treatment, the postoperative pulmonary arterial pressure in most patients was decreased below 40 mmHg [6]. Even in a patient with a right-to-left shunt, the pulmonary artery pressure decreased to the normal range postoperatively [8]. Such outcomes are better than predicted based on the pathophysiology of this complex anomaly.

Among 29 patients for whom information was available, 22 patients (75.9%) experienced postoperative complications during the follow-up period; 20 of 26 patients in the one-stage surgery group (76.9%) had a postoperative complication compared to 2 of 3 (66.7%) in the two-stage surgery group (x^2^ = 0, *p* = 1.0). The high rate of postoperative complications indicates the value of long-term follow-up. Complications mainly presented as great vessel narrowing and residual defects. Most patients (58.6%) suffered from RPA stenosis as a result of either compression by the aorta or excessive tension on the pulmonary anastomosis [53]. In the reports we reviewed, 44.8% and 3.4% of the patients had stenosis at the aortic arch and residual APW, respectively, which accords with the findings of a previous study [6]. Reinterventions were required in 13 of 27 patients (48.1%) during the follow-up period: 13 of 25 patients in the one-stage group underwent reinterventions, while neither of the 2 patients in the two-stage surgery group underwent reintervention (x^2^ = 2.01, *p* = 0.48). Reinterventions for stenosis at the aortic arch were performed in 9 patients, including surgical repair of residual arch obstruction in 3 and balloon angioplasty of the aortic arch in 6. Reinterventions for RPA stenosis were performed in 7 patients, including surgical repair of RPA obstruction in 5 and balloon angioplasty of the RPA in 3 (one of the patients underwent both surgical repair and balloon angioplasty of the RPA). Analyzed by binary logistic regression, the major morphologic factors (morphology of aortic artery, IAA type, APW type, patent ductus arteriosus type, original RPA site, and type of surgery) were not independent predictors of complications and reintervention after surgery.

In conclusion, Berry syndrome is a rare but well-identified and surgically correctable anomaly. Echocardiography can provide adequate diagnostic information and more than half of the patients diagnosed before 3 months old. Most patients with Berry syndrome presented with type II APW, type A IAA, PDA, and the RPA usually arose from the aorta. Pulmonary hypertension was common in Berry syndrome patients and the postoperative pulmonary arterial pressure usually decreased below 40 mmHg. We found 50.0% patients underwent surgery before 1 month old and no differences in mortality within a month postoperatively or survival time within the one-year follow-up period between different types of surgery. Some patients would experience postoperative complications and reinterventions were required. In addition, we discussed the possible reasons why unoperated patients might achieve long-term survival and whether it is necessary to use prostaglandin prophylactically before surgery. However, the major limitation of this review is that the data analysis was based on the incomplete information available. There is a publication bias since an unknown number of patients died early without a diagnose or being offered surgical repair. Meantime, long-term survivors included in cohort studies describing patients with cardiac anomalies (e.g. IAA, APW, AORPA and PDA) may not be recognized in our literature search. Therefore, the results of this review should be treated with caution. Patients with Berry syndrome should be followed up for longer periods to better characterize long-term outcomes.

## Supplementary Information

**Additional file 1**. Literature review of 89 cases of Berry syndrome from 1982 to June 2019. The following clinical data were extracted and reviewed: sex, presence of clinical symptoms, premature birth, age at diagnosis, diagnostic methods, anomalies recognized by echocardiography, anatomic features of the cardiac anomalies (morphology of aortic artery, APW type, type of ductus arteriosus, and original site of RPA), associated cardiac and extracardiac anomalies, chromosomal abnormalities, age at surgery, types of surgery, postoperative details (postoperative hospitalization, postoperative follow-up time, postoperative complications, and reintervention after surgery), outcome, cause of death, preoperative and postoperative pulmonary artery pressure, and the presence of a massively dilated vascular sac.

## Data Availability

All data generated or analysed during this study are included in this published article [and its Additional file 1].

## Abbreviations

APW: Aortopulmonary window
AORPA: Aortic origin of the right pulmonary artery
IAA: Interrupted aortic arch
HAA: Hypoplastic aortic arch
CoA: Coarctation of the aorta
RPA: Right pulmonary artery
PDA: Patent ductus arteriosus
LPA: Left pulmonary artery
